# A New ICE*clc* Subfamily Integrative and Conjugative Element Responsible for Horizontal Transfer of Biphenyl and Salicylic Acid Catabolic Pathway in the PCB-Degrading Strain *Pseudomonas stutzeri* KF716

**DOI:** 10.3390/microorganisms9122462

**Published:** 2021-11-29

**Authors:** Jun Hirose, Takahito Watanabe, Taiki Futagami, Hidehiko Fujihara, Nobutada Kimura, Hikaru Suenaga, Masatoshi Goto, Akiko Suyama, Kensuke Furukawa

**Affiliations:** 1 Department of Applied Chemistry, Faculty of Engineering, University of Miyazaki, Miyazaki 889-2192, Japan; 2 Research Institute for Sustainable Humanosphere, Kyoto University, Uji 611-0011, Japan; takahito@rish.kyoto-u.ac.jp; 3 Education and Research Center for Fermentation Studies, Faculty of Agriculture, Kagoshima University, Kagoshima 890-0065, Japan; futagami@agri.kagoshima-u.ac.jp; 4 Department of Food and Fermentation Sciences, Faculty of Food and Nutrition Sciences, Beppu University, Beppu 874-8501, Japan; fujihara@nm.beppu-u.ac.jp (H.F.); aksuyama@nm.beppu-u.ac.jp (A.S.); kfurukaw@nm.beppu-u.ac.jp (K.F.); 5 Bioproduction Research Institute, National Institute of Advanced Industrial Science and Technology (AIST), Tsukuba 305-8566, Japan; n-kimura@aist.go.jp; 6 Cellular and Molecular Biotechnology Research Institute, National Institute of Advanced Industrial Science and Technology (AIST), Tokyo 135-0064, Japan; suenaga-hikaru@aist.go.jp; 7 Faculty of Agriculture, Saga University, Saga 840-8502, Japan; mgoto@cc.saga-u.ac.jp

**Keywords:** horizontal gene transfer, ICE*bph-sal*, ICE*clc*, ICE*_XTD_*, integrative and conjugative elements, polychlorinated biphenyls, *Pseudomonas stutzeri*, salicylic acid

## Abstract

Integrative and conjugative elements (ICEs) are chromosomally integrated self-transmissible mobile genetic elements. Although some ICEs are known to carry genes for the degradation of aromatic compounds, information on their genetic features is limited. We identified a new member of the ICE*clc* family carrying biphenyl catabolic *bph* genes and salicylic acid catabolic *sal* genes from the PCB-degrading strain *Pseudomonas stutzeri* KF716. The 117-kb ICE*_bph-sal_*KF716 contains common core regions exhibiting homology with those of degradative ICE*clc* from *P. knackmussii* B13 and ICE*_XTD_* from *Azoarcus* sp. CIB. A comparison of the gene loci collected from the public database revealed that several putative ICEs from *P. putida* B6-2*, P, alcaliphila* JAB1, *P. stutzeri* AN10*,* and *P. stutzeri* 2A20 had highly conserved core regions with those of ICE*_bph-sal_*KF716, along with the variable region that encodes the catabolic genes for biphenyl, naphthalene, toluene, or phenol. These data indicate that this type of ICE subfamily is ubiquitously distributed within aromatic compound-degrading bacteria. ICE*_bph-sal_*KF716 was transferred from *P. stutzeri* KF716 to *P. aeruginosa* PAO1 via a circular extrachromosomal intermediate form. In this study, we describe the structure and genetic features of ICE*_bph-sal_*KF716 compared to other catabolic ICEs.

## 1. Introduction

Integrative and conjugative elements (ICEs) are mobile genetic elements of bacteria that are excised from the chromosome, transferred to other bacteria via conjugation, and reintegrated into the chromosome. They often carry cargo genes involved in antibiotic resistance, pathogenicity, heavy metal resistance, nitrogen fixation, or aromatic ring catabolism to impart beneficial traits to bacteria [1]. To date, a limited number of ICEs are known to carry cargo genes for the catabolism of aromatic polluting compounds, of which ICE*clc* is one of the best-characterized [2]. ICE*clc* contains cargo genes that encode the *ortho-*cleavage of chlorocatechol (*clc* genes) and aminophenol catabolism (*amn* genes). This element was originally identified in the 3-chlorobenzoic acid-degrading bacterium *Pseudomonas knackmussii* B13. Almost identical copies have been found on the chromosome of *Burkholderia xenovorans* LB400 (designated ICE*clc*-LB400) [3] and *P. aeruginosa* JB2 (ICE*clc*-JB2) [4]. The structure of ICE*clc* was compared with other conserved ICEs from five bacterial strains, but none of them contained a gene encoding the aromatic ring degradation pathway [5]. ICE*_XTD_* from *Azoarcus* sp. CIB is another member of the ICE*clc* family, which is well characterized in terms of the function of cargo genes and the transferability by conjugation [6]. A notable feature of ICE*_XTD_* is that it carries gene clusters for both aerobic and anaerobic degradation of xylene and toluene.

The *bph* genes, which are responsible for the co-metabolic degradation of polychlorinated biphenyls (PCBs), are widely distributed among both Gram-negative and Gram-positive bacteria [7]. Some *bph* genes are known to be horizontally transferred via mobile genetic elements. The first reported ICE carrying *bph* genes is Tn*4371*, found in the chromosome of the Gram-negative bacterium *Cupriavidus oxalacticus* A5 [8], with a total length of 61.8 kb. The chromosome of the Gram-negative bacterium *Acidovorax* sp. strain KKS102 contains ICE_KKS102_*4677*, which belongs to the Tn*4371* family [9]. ICE_KKS102_*4677* is known to transfer to a wide variety of bacteria across species and genera via a circular intermediate. Recently, we reported the entire genomes of ten PCB-degrading bacteria isolated from biphenyl-contaminated soil in Kitakyushu, Japan [10]. Among them, we detected ICEs carrying the *bph* gene from nine strains. ICE*_bph_*KF708 from *Cupriavidus basilensis* KF708 and ICE*_bph_*KF712 from *Comamonas testosteroni* KF712 are Tn*4371* type ICEs, where ICE*_bph_*KF708 is almost identical to ICE_KKS102_*4677*. A 483-kb plasmid pKF715A carrying the *bph* gene and salicylic acid catabolic *sal* gene was detected from *P. putida* KF715, which could be transferred and integrated into the chromosome of *P. putida* AC30 or KT2440, and then maintained as ICE*_bph-sal_*KF715 [11]. Six *Pseudomonas* strains (*P. abietaniphila* KF701, *P. aeruginosa* KF702, *P. putida* KF703, *P. furukawaii* KF707 (formerly *P. pseudoalcaligenes* KF707), *P. toyotomiensis* KF710, and *P. stutzeri* KF716) carry an ICE*_bph-sal_* element with sizes ranging from 117 kb to 130 kb, and integrate at the 3′ end of the tRNA-Gly(CCC) gene. It is obvious from their highly conserved sequences that ICE*_bph-sal_*s were generated via horizontal gene transfer. These ICEs carrying *bph* genes play an important role in the degradation of PCBs in the environment. Precise information on their structure and function will be important to understand the adaptation of their host strains to environmental niches and to design bioremediation processes using PCB-degrading bacteria. Here, we investigated the gene structure of ICE*_bph-sal_*KF716 in comparison with other catabolic ICEs and demonstrated that ICE*_bph-sal_*KF716 can be transferred via a circular intermediate form.

## 2. Materials and Methods

### 2.1. Bacterial Strains and Culture Conditions

*Pseudomonas stutzeri* KF716 (NBRC 110668), which has the ability to utilize biphenyl (Bph^+^) and salicylic acid (Sal^+^), was isolated from the soil in Kitakyushu, Japan [10]. *P. aeruginosa* PAO1(NBRC 106052), which has kanamycin resistance (Km^R^), was obtained from the Biological Resource Center of the National Institute of Technology and Evaluation (NBRC, Tokyo, Japan). For the growth of *Pseudomonas* strains, a basal salt medium containing (in grams per liter) K_2_HPO_4_, 4.3; KH_2_PO_4_, 3.4; (NH_4_)_2_SO_4_, 2.0; MgCl_2_·6H_2_O, 0.34; MnCl_2_·4H_2_O, 0.001; FeSO_4_·7H_2_O, 0.0006; CaCl_2_·2H_2_O, 0.026; and Na_2_MoO_4_·2H_2_O, 0.002 (pH 7.0) was used. The bacterial strains were grown by shaking at 120 rpm at 30 °C. For DNA isolation or freezing stock preparation, Luria–Bertani (LB) medium (Bacto Tryptone, 10 g; yeast extract, 5 g; and NaCl, 10 g/L, pH 7.0) was used.

### 2.2. Sequence Annotation and Computational Analysis

The sequence of ICE*_bph-sal_*KF716 was determined as previously reported [10,12]. The complete nucleotide sequence of ICE*_bph-sal_*KF716 has been deposited in DDBJ/ENA/GenBank under accession no. LC469614. The sequences were annotated using the NCBI Prokaryotic Genome Annotation Pipeline (PGAP) [13] and Rapid Annotations using Subsystems Technology (RAST) server v.2.0 [14]. The coding genes were identified using BLAST and BLASTX searches [15]. Sequence comparisons were performed using EasyFig v.2.1 [16], and a map was generated using the drawGeneArrows3 program (http://www.ige.tohoku.ac.jp/joho/labhome/tool.html, accessed on 28 November 2021). GC content and identity between genes were calculated using GENETYX version 15 (Genetyx Co. Ltd., Tokyo, Japan).

### 2.3. Detection of Target DNA by Polymerase Chain Reaction

Genomic DNA was extracted using a Genomic-tip 20/G (Qiagen, Hilden, Germany), according to the manufacturer’s instructions. Amplification of genes was performed in a thermal cycler GeneAmp PCR System 9700 (Applied Biosystems, Foster City, CA, USA) and PCR conditions were performed in a 25 μL reaction mix containing 12.5 μL Gene RED PCR Mix Plus (Nippon Gene Co. Ltd., Toyama, Japan) at 94 °C for 5 min, followed by 30 cycles of 94 °C for 45 s, 62 °C for 45 s, and 72 °C for 60 s, with a final extension of 5 min at 72 °C. See Table S1 for primer sequences. PCR products were detected by agarose gel electrophoresis according to a standard procedure. The sequences of PCR products were determined using the BigDye Terminator v3.1 Cycle Sequencing Kit and an ABI PRISM 3500 Genetic Analyzer.

### 2.4. Conjugal Transfer of ICE

Transfer of the Bph^+^ phenotype by conjugation into the recipient cells was carried out through filter mating. Donor and recipient cells were grown overnight in LB agar medium, and both cultures were suspended in 1 mL of LB broth. The cell suspensions (0.5 mL each) were mixed and placed on a nitrocellulose filter (0.45 μm, Merck Millipore, Bedford, MA, USA), and placed on an LB agar plate at 30 °C for 16 h. After incubation, the cells on the filter were suspended in sterilized saline, further diluted, and inoculated onto basal salt medium plates containing 30 μg of kanamycin, providing solid biphenyl in the inverted lid to select transconjugants. Conjugative transfer frequencies were calculated as the number of transconjugant cells per number of donor bacterial cells present in each mating.

## 3. Results and Discussion

### 3.1. Detection of ICE_bph-sal_KF716

**Figure 1:**
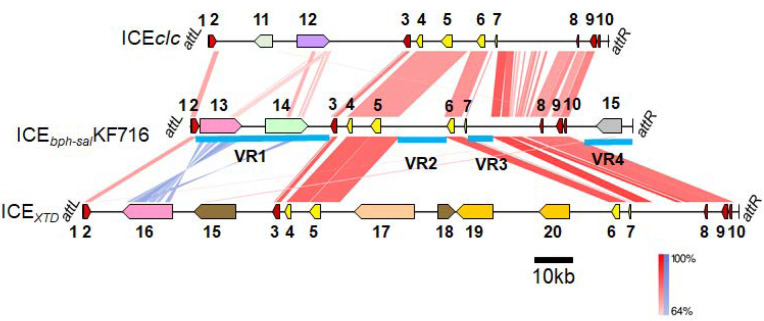
Gene map of ICE*clc*, ICE*_bph-sal_*KF716, and ICE*_XTD_*. Regions with nucleotide identity above 64% are connected by red (forward) or blue (reverse) windows using a color intensity gradient based on identity scores of BLASTn comparison. 1: tRNA-Gly (partial); 2: *int* genes; 3: *traI* gene; 4: *traG* gene; 5: VirB4 components of the type IV secretory pathway; 6: VirD4 component of the type IV secretory pathway; 7: *pilL*; 8: *inrR*; 9: *parB*; 10: *parA*; 11: *clc* genes; 12: *amn* genes; 13: *bph* genes; 14: *sal* genes; 15: putative multi-drug efflux pumps; 16: *tod* genes, 17: *mdb* genes; 18: putative benzoate transporter; 19: *bss* genes; 20: *bbs* genes.

**Figure 2:**
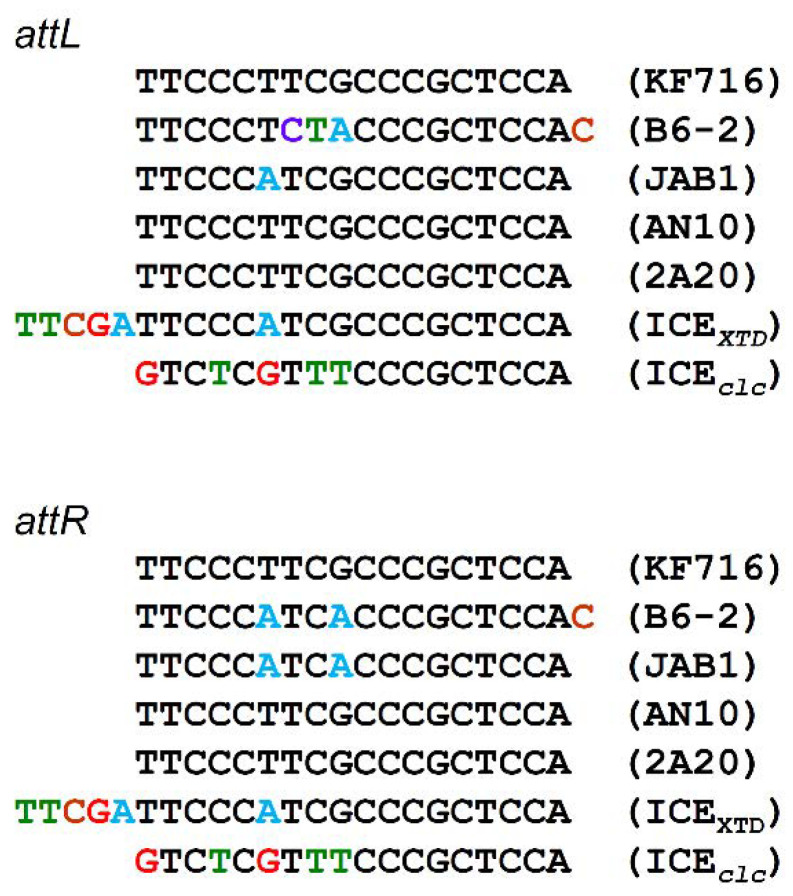
Direct repeat sequences flanking ICE*_bph-sal_*, ICE*clc*, ICE*_XTD,_* and other putative ICEs. The *attL* and *attR* correspond to the sequence of the 5’ and 3’ termini of the respective ICE.

A detailed analysis of the genome sequence of *P. stutzeri* KF716 revealed a genomic island of 117,300 bp (Figure 1), named ICE*_bph-sal_*KF716. ICE*_bph-sal_*KF716 is located at the 3’ end (*attB* site) of tRNAGly(CCC). The left end (*attL*) of ICE*_bph-sal_*KF716 is formed by 18 bp of tRNA-Gly (TTCCCTTCGCCCGCTCCA), and the right end (*attR*) is formed by a repetition of these 18 bp (Figure 2). We previously confirmed that this 18 bp direct repeat sequence is located on the border of the conserved ICE region and the non-conserved region of the chromosome [10]. The complete sequence of ICE*_bph-sal_*KF716 was submitted to the PGAP and RAST pipeline for annotation, and 112 ORFs were identified. Annotation was refined manually and compared with pKF715A [11], ICE*clc* [2], and ICE*_XTD_* [6]. ICE*_bph-sal_*KF716 is a mobile genetic element that shares a core region with ICE*clc* and ICE*_XTD_*, which have been shown to impart aromatic compound degradation genes to bacteria. The genetic maps of ICE*_bph-sal_*KF716, ICE*clc,* and ICE*_XTD_* are shown in Figure 1, while their *attL* and *attR* are shown in Figure 2. The genes encoded by ICE*_bph-sal_*KF716 are listed in Table S2.

### 3.2. Core Region of ICE_bph-sal_KF716

The *attL* gene was found to be followed by a phage-related integrase (*int*) gene, which shares an identity with that of ICE*clc* or ICE*_XTD_* carrying catabolic genes of aromatic compounds. ICE*_bph-sal_*KF716 contains core regions that are significantly similar to the core regions of ICE*clc* [2] and ICE*_XTD_* [6] (Figure 1). The ORFs in the core region of ICE*_bph-sal_*KF716 were 55–83% identical to those of ICE*clc,* and 57–93% identical to those of ICE*_XTD_*. The ORFs display the same genetic order in the ICE*_bph-sal_*KF716 element, and belong to the type IV secretion system (T4SS). Three factors have been recognized as important for DNA transfer by T4SS in Gram-negative and Gram-positive bacteria. These are: murein hydrolase [17], which is involved in controlled local degradation of the peptidoglycan, making space for the formation of a mating channel; the VirB4 ATPase, which provides energy for the translocation: and the VirD4 coupling protein, which links the DNA transfer intermediate to the mating channel [18]. In the ICE*_bph-sal_*KF716 element, we identified the *virB4* gene (KF716ICE_480; the locus tags are listed in Table S2), *virD4* gene (KF716ICE_650), and putative murein hydrolase (KF716ICE_670).

The *parA* and *parB* genes (KF716ICE_960, KF716ICE_940) encoding the replication partition proteins present 100 kb and 99 kb downstream of the *attL* site, respectively, were proposed to act as a stabilization system for the maintenance of mobile elements in the bacterial genomes [19]. Near *parB,* there is a gene encoding putative integrase regulator (KF716ICE_890) a homolog of InrR involved in the regulation of the expression of integrase on ICE*clc* [20]. Relaxase (*traI*, KF716ICE_370) which binds and nicks excised circular ICE at the origin of the transfer [21], TraG protein (*traG*, KF716ICE_410) [22], a component of the mating pair formation system, and pilin protein (*pilL*, KF716ICE_700) were also identified in the core region of ICE*_bph-sal_*KF716.

The homology of the integrase-encoding *int* gene between ICE*_bph-sal_* and ICE*clc* was lower (66%) than that in the core region. The integration site of ICE*_bph-sal_* was tRNA-Gly(CCC), whereas that of ICE*clc* was different in tRNA-Gly(GCC). In contrast, the homology of the *int* gene between ICE*_XTD_* and ICE*_bph-sal_*KF716 was 70%; the integration site of ICE*_XTD_* was tRNA-Gly(CCC), which was identical to that of ICE*_bph-sal_*KF716. The sequence of *attL* and a*ttR* at the end of ICE was five bases different between ICE*clc* and ICE*_bph-sal_*KF716, and one base difference between ICE*_XTD_* and ICE*_bph-sal_*KF716 (Figure 2). The amino acid sequences of these ICE integrases may reflect different recognition of the integration site.

### 3.3. Variable Region of ICE_bph-sal_KF716

**Figure 3:**
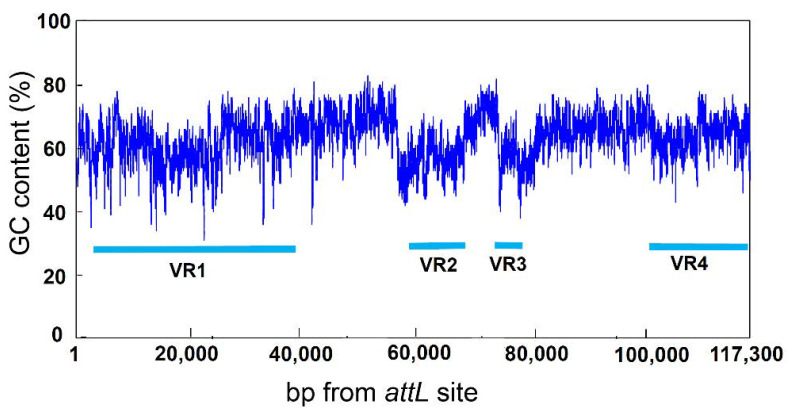
GC content of ICE*_bph-sal_*KF716. The horizontal bars labeled VR1 to VR4 represent four variable regions.

ICE*_bph-sal_*KF716 contains at least four variable regions (VR1–VR4) that are deficient in ICE*clc* and ICE*_XTD_* (Figure 1). Variable region 1 (VR1) located near the *attL* site contains the biphenyl catabolic *bph* gene cluster with a total length of 11.2 kb, as well as a salicylate catabolic *sal* gene cluster with a total length of 11.5 kb. The *bph* gene cluster was found to be located just downstream of the *int* gene, followed by the *sal* gene cluster approximately 6 kb further downstream. The *bph* and *sal* genes encode for the degradation of biphenyl and salicylic acid to TCA cycle intermediates via an initial oxygenation step, followed by a *meta*-cleavage pathway. As shown in Figure 1, the *bph* gene cluster in VR1 shares 59–79% identity at the nucleotide sequence level with the toluene catabolic *tod* gene cluster located in the variable region of ICE*_XTD_* in the opposite direction. The relationship between the *bph* gene of *P. furukawaii* KF707 and the *tod* gene of *P. putida* F1 has been reported in our previous paper [23]. The two variable regions (VR2 and VR3) are located at the midst of ICE*_bph-sal_*KF716. The GC content of these regions was lower than that of the other regions (Figure 3), and these ORFs (from KF716ICE_570 to KF716ICE_630 and from KF716ICE_710 to KF716ICE_740) were encoded in the opposite direction compared with ORFs in the surrounding core regions (Table S2), suggesting that these regions are inserted from the other genetic elements or chromosomes through horizontal gene transfer. VR2 contained ORFs coding for several hypothetical proteins with unknown functions, whereas VR3 is likely to be an insertion sequence since it includes an ORF identified as transposase (KF716ICE_720). The other variable region (VR4) located near the *attR* site contained the gene cluster coding for a putative ABC-type multi-drug efflux pump (from KF716ICE_1060 to KF716ICE_1110), with a total length of 6.6 kb that is deficient in ICE*clc* and ICE*_XTD_*. The substrate of this transporter was not identified due to the lack of reliable homologous genes whose functions have been elucidated.

### 3.4. Comparison of ICE_bph-sal_KF716 and Other Putative ICEs

A search of the public database using BLAST revealed that an almost identical core region to that of ICE*_bph-sal_*KF716 was found in other putative ICEs. The identities were higher than those of ICE*clc* or ICE*_XTD_*. Putative ICEs were identified from the genome sequences of *P. putida* B6-2 (accession number: NZ_CP015202) [24,25], *P. alcaliphila* JAB1 (CP016162) [26], *P. stutzeri* AN10 (NC_018028) [27], and *P. stutzeri* 2A20 (KT935509) [28]. They are flanked by directed repeat sequences corresponding to the *attL* and *attR* sites (Figure 2).

**Figure 4:**
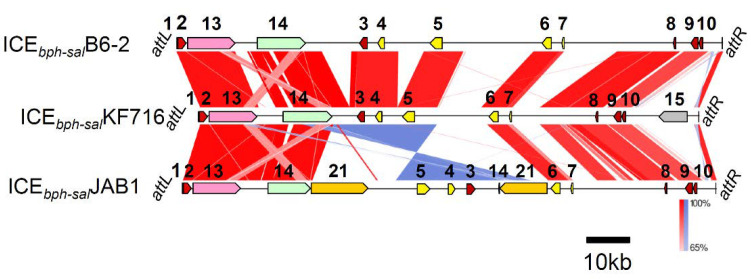
Comparison of ICE*_bph-sal_*KF716 and putative ICE*_bph-sal_* regions in *P. putida* B6-2 (NZ_CP015202) and *P. alcaliphila* JAB1 (CP016162). Regions with nucleotide identity above 65% are connected following the legend to Figure 1. The number of the genes are defined as shown in Figure 1 aside from the *bza* gene (21).

The putative ICE from *P. putida* B6-2 (tentatively designated as ICE*_bph-sal_*B6-2) has the closest relationship with ICE*_bph-sal_*KF716; it carries a *bph-sal* gene cluster that shares 91–100% identity with that of ICE*_bph-sal_*KF716, as well as highly conserved core regions (Figure 4). *P. putida* B6-2 is capable of degrading various polycyclic aromatic hydrocarbons [25]. Biphenyl (*bph*), salicylic acid (*sal*), and ferulic acid (*fcs, ech*), as well as downstream benzoic acid (*ben*) and protocatechuic acid (*pca*) catabolic gene clusters were identified on the genome sequence of *P. putida* B6-2, of which *bph* genes and *sal* genes are located in the ICE (Figure 4). The other putative ICE integrated in the genome of *P. alcaliphila* JAB1 (tentatively designated as ICE*_bph-sal_*JAB1) is the second-closest ICE to ICE*_bph-sal_*KF716; in this, the *bph-sal* gene cluster and benzoate catabolic *bza* gene cluster are included in the variable region (Figure 4). It is likely that inversion in the variable region, together with a part of the core region, occurred in the ICE*_bph-sal_*JAB1. In addition, *sal*:*bza* and *bza*:*sal* fusion gene clusters were found, in which parts of the *sal* genes and the *bza* genes were replaced with one another. This inversion at the *sal-bza* locus was also detected in ICE*_bph-sal_*KF702 of *P. aeruginosa* KF702, as described in our previous paper [10]. In addition to the well-characterized Tn*4371* and ICE_KKS102_4677, ICEs that carry the *bph* gene cluster included ICE*_bph-sal_*KF701, ICE*_bph-sal_*KF702, ICE*_bph-sal_*KF703, ICE*_bph-sal_*KF707, and ICE*_bph-sal_*KF710 from five biphenyl/PCB-degrading strains isolated from Kitakyushu, Japan [10]. ICE*_bph-sal_*KF716 shares a core region and *bph-sal* catabolic genes with these ICE*_bph-sal_* elements, but lacks the *bza* gene, which encodes the benzoic acid degradation pathway. The comparison of the overall structure revealed that ICE*_bph-sal_*B6-2 from *P. putida* B6-2, and ICE*_bph-sal_*JAB1 from *P. alcaliphila* JAB1 are also members of the ‘ICE*_bph-sal_* family’ (Figure 4). In particular, ICE*_bph-sal_*B6-2 is more closely related to ICE*_bph-sal_*KF716 as it lacks the *bza* gene. Since *P. alcaliphila* JAB1 was isolated in the Czech Republic, and *P. stutzeri* KF716 was isolated from Japan [10], it appears that ICE*_bph-sal_*s are globally distributed. 

**Figure 5:**
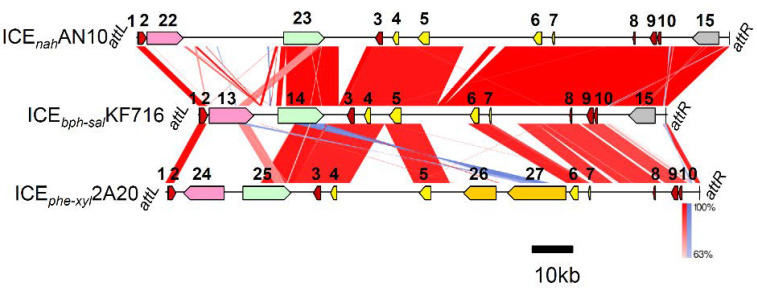
Comparison of ICE*_bph-sal_*KF716 (accession number of genome sequence: LC469614) and putative ICEs in *P. stutzeri* AN10 (NC_018028) and *P. stutzeri* 2A20 (KT935509). Regions with nucleotide identity above 63% are connected following the legend to Figure 1. 22, *nah* upper operon; 23, *nah* lower (*sal*) operon; 24, *tou* genes; 25, *phe* genes; 26, *xyl* upper operon; 27, *xyl* lower operon. Other number of the genes are defined following the legend to Figure 1.

A BLAST search identified the other putative ICEs carrying a highly conserved core region with that of ICE*_bph-sal_*KF716 (Figure 5). The genome of *P. stutzeri* AN10 contained the putative ICE (tentatively designated ICE*_nah_*AN10) carrying the naphthalene catabolic *nah* genes [29,30] that share more than 70–90% homology with the *nah* operon on plasmid NAH7 at the nucleotide sequence level. The aerobic degradation pathway of naphthalene consists of an upper pathway that transforms naphthalene to salicylic acid and pyruvic acid, and a lower pathway that transforms salicylic acid to TCA cycle intermediates. The *nah* lower operon coding for the lower pathway of ICE*_nah_*AN10 shares 97–100% identity with the *sal* genes of ICE*_bph-sal_*KF716. In this context, it is likely that the *nah* upper operon is replaced by the *bph* genes in ICE*_bph-sal_*KF716, and, conversely, the *bph* genes are replaced by the *nah* upper operon in ICE*_nah_*AN10. It has been confirmed that many putative mobile protein genes are present on ICE*_bph-sal_*s [10], which may act to replace the *bph* genes and the *nah* upper operon. The part of ICE*_nah_*AN10, including the core region and genes coding for putative multi-drug efflux pumps, is perfectly identical (100%) to that of ICE*_bph-sal_*KF716, showing a very close relationship between the two ICEs. In fact, our previous paper described that the *nah* lower operon of *P. stutzeri* AN10 and the *sal* gene of *P*. *furukawaii* KF707 are highly conserved [31].

The putative ICE from *P. stutzeri* 2A20 (tentatively designated lCE*_phe-xyl_*2A20) carried the *tou* genes coding for multicomponent toluene monooxygenases, *phe* genes coding for phenol *meta*-cleavage pathway, and *xyl* upper and lower operons coding for toluene-xylene catabolic genes [28] in the variable regions. The core regions of ICE*_phe-xyl_*2A20, including partial *phe* genes, overlapped with the *sal* gene share 91–97% identity with those of ICE*_bph-sal_*KF716. A comparison of the variable regions of ICE*_phe-xyl_*2A20 and ICE*_bph-sal_*KF716 strongly indicates that the substitution occurred between the biphenyl catabolic *bph* genes and toluene monooxygenase *tou* genes together with the part of the *phe* genes. Although ICE*_nah_*AN10 and ICE*_phe-xyl_*2A20 do not carry *bph* genes, the evolutionary relationship of ICE*_bph-sal_*KF716 with ICE*_nah_*AN10 or ICE*_phe-xyl_*2A20 was closer than that of ICE*clc* or ICE*_XTD_*, as judged from the nucleotide sequence identity level. Their homologies of the core regions with ICE*_bph-sal_* were higher (77–100% identity between ORFs) than those of ICE*clc* and ICE*_XTD_*, indicating that these ICEs are more closely related to ICE*_bph-sal_*KF716. The identity of ICE*_bph-sal_*KF716 with ICE*_nah_*AN10 or ICE*_phe-xyl_*2A20 varies depending on the regions, where major parts including integrase (*int*) gene were highly conserved (90–100% identity), as shown in Figure 5. Although the variable regions among ICE*_bph-sal_*KF716, ICE*_nah_*AN10, and ICE*_phe-xyl_*2A20 are different, they commonly possess genes for aromatic compound *meta-*cleavage pathways. ICE*_bph-sal_*KF716, ICE*_nah_*AN10, and ICE*_phe-xyl_*2A20 are all ICEs found in *P. stutzeri*. This species is known to exhibit phenotypical diversity in the ecosystem and can adapt to the environmental niche [32], suggesting that it is particularly preferable as a host for this ICE subfamily.

### 3.5. Excision and Formation of Circular Intermediate Form of ICE_bph-sal_KF716

**Figure 6:**
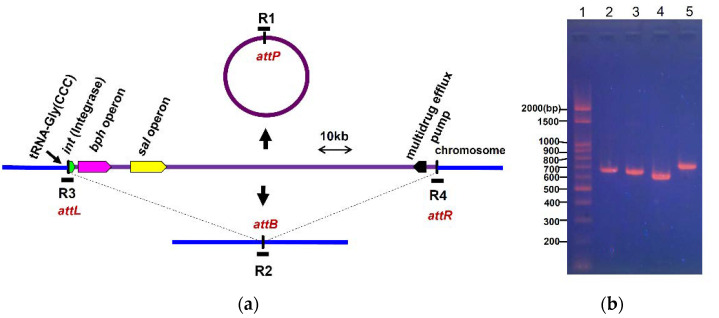
(**a**) Schematic representation of formation of circular form and integrated form of ICE*_bph-sal_*KF716. The horizontal bars labeled R1 to R4 are the DNA regions amplified by PCR. Primers attL1 and attR1 (sequences given in Table S1) were used to amplify R1 including *attP* site of ICE*_bph-sal_*KF716. Primers attL1 and attL2 were used to amplify R2 including *attB* site. Primers attL1 and attL2 were used to amplify R3 including *attL* site. Primers attR1 and attR2 were used to amplify R4 including *attR* site. (**b**) Detection of circular form and integrated form of ICE*_bph-sal_*KF716 in wild type *P. stuzeri* KF716 by PCR. Lane 1, DNA ladder marker; Lane 2, R1 (*attP*); Lane 3, R2 (*attB*); Lane 4, R3 (*attL*); Lane 5, R4 (*attR*).

ICEs integrated into the chromosome can be excised from the chromosome to produce a circular form, and the host genome was repaired upon excision (Figure 6a). Attempts were made to detect the extrachromosomal circular form of ICE*_bph-sal_*KF716 from total DNA isolated from *P. stutzeri* KF716 grown on biphenyl. The amplicons corresponding to the *attP* site (R1 in Figure 6a) of excised circular forms of ICE*_bph-sal_*KF716, *attB* sites (R2) of excised closed forms of chromosome, and *attL*/*attR* sites (R3 and R4) of the integrated form were detected using PCR (Figure 6b). It has been reported that tRNA-Gly(CCC) locus is the insertion site of ICE*_bph-sal_*KF716 [10]. DNA sequences of these amplicons matched the expected sequences compared to the total genome sequence of *P. stutzeri* KF716 [12].

### 3.6. Transfer and Integration of ICE_bph-sal_KF716

**Figure 7:**
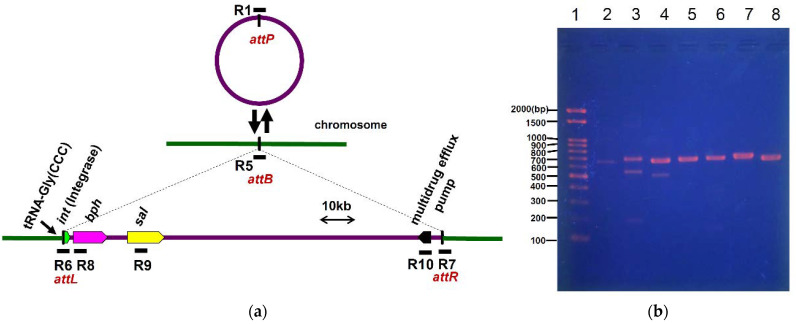
(**a**) Schematic representation of the integration of ICE*_bph-sal_*KF716 into chromosome of *P. aeruginosa* PAO1. The horizontal bars labeled R5 to R10 are the DNA regions amplified by PCR. Primers attL3 and attR3 (sequences were given in Table S1) were used to amplify R5 including the *attB* site of the recipient strain. Primers attL1 and attL3 were used to amplify R6 including the *attL* site. Primers attR1 and attR3 were used to amplify R7 including the *attR* site. Primers bphL1 and bphR1 were used to amplify R8 including the *bph* gene. Primers salL1 and salR1 were used to amplify R9 including the *sal* gene. Primers MDL1 and MDR1 was used to amplify R10 including the putative gene for multi-drug efflux pump. (**b**) Detection of integrated from of ICE*_bph-sal_*KF716 in transconjugant *P. aeruginosa* PAO1 by PCR. Lane 1, DNA ladder marker; Lane 2, R1 (*attP*); Lane 3, R5 (*attB*); Lane 4, R6 (*attL*); Lane 5, R8 (*bph*); Lane 6, R9 (*sal*); Lane 7, R10 (multi-drug efflux pump); Lane 8, R7 (*attR*).

To demonstrate the autonomous intercellular transfer of ICE*_bph-sal_*KF716, we performed mating experiments using *P. stutzeri* KF716 as a donor strain. *P. aeruginosa* PAO1 was used as the recipient strain because it carries Km^R^ phenotype, and forms a green colony, which enables it to be distinguished from the donor strain. The donor strain was mated with the recipient strain, and transconjugants were selected based on their Km^R^ and Bph^+^ phenotypes. We observed the appearance of transconjugants, *P. aeruginosa* PAO1 acquiring Bph^+^, Sal^+^ and Km^R^ phenotypes. The transfer frequencies (transconjugants per donor cell) ranged from 6.2 × 10^−8^ to 1.3 × 10^−6^, with 3.3 × 10^−7^ average in eight replicates. The transconjugants grew in a liquid medium containing biphenyl and salicylic acid as the sole source of carbon (data not shown). To confirm the identity of the transconjugant cells, PCR was performed with genomic DNA using primers 27F and 907R that amplify the 16S ribosomal DNA (16S rDNA), and the DNA sequence of the amplicon matched the 16S rDNA of the recipient strain. The amplicons corresponding to the *attL*/*attR* site (R6 and R7 in Figure 7a) of the integrated form of ICE*_bph-sal_*KF716 were detected from at least three PAO1 transconjugants. (Figure 7b). DNA sequencing these amplicons derived from the integrated form of ICE*_bph-sal_*KF716 in the transconjugants confirmed *attB* site at the 3′ end of tRNA-Gly(CCC) overlapping with sequence position 797,721 to 797,738 in genome sequence of *P. putida* PAO1 (accession number: NC_002516) [33]. The amplicons corresponding to the *attP* site (R1) of excised circular forms of ICE*_bph-sal_*KF716 and the *attB* site (R5) of excised closed form of chromosome were also detected from the transconjugants. DNA sequences of these amplicons matched the expected sequences compared to the *attP* site of ICE*_bph-sal_*KF716 or its insertion site on the *P. aeriginosa* PAO1 chromosome. At least two different sized amplicons were observed by electrophoresis (Figure 7b, Lane 3 and Lane 4) and by DNA sequencing when trying to obtain a fragment of R5 corresponding to the *attB* site or R6 corresponding to the *attL* site, respectively. This result indicates heterogeneity in the transconjugants, probably due to partial excision of ICE*_bph-sal_*KF716 from chromosome. It has been reported that the transfer efficiency of ICE*clc* is extremely high (1.4 × 10^−2^) [20], whereas that of ICE_KKS102_*4677* is extremely low (5.8 × 10^−10^) [9]. The transfer efficiency of ICE*_bph-sal_*KF716 was approximately 3.3 × 10^−7^ in the intermediate of ICE*clc* and ICE_KKS102_*4677* and comparable with that of ICE*_XTD_*. The factors that govern the transfer efficiency of these ICEs remain to be elucidated, however many factors seem to involve the donor and recipient strains.

## 4. Conclusions

In this study, we investigated the structure and transfer of ICE*_bph-sal_*KF716, which is integrated into the chromosome of the biphenyl/PCB-degrading bacterium *P. stutzeri* KF716. Comparison with putative ICEs from other related *Pseudomonas* strains suggested the existence of a new ICE*clc* subfamily that shares nearly identical core regions. Since ICE*_bph-sal_*KF716 is the first member of this subfamily found, ICE*_bph-sal_*KF716 represents the ICE*clc* subfamily which is involved in the horizontal transfer of various catabolic genes among *Pseudomonas* strains. The results presented in this study will provide new insights into the evolution of ICEs in the process of adaptation to environmental niches, as well as a basis for designing bioremediation processes using PCB-degrading bacteria.

## Data Availability

The data presented in this study are available on request from the corresponding author.

## Supplementary Materials

The following are available online at https://www.mdpi.com/article/10.3390/microorganisms9122462/s1, Table S1: PCR primers used in this study, Table S2: Genes encoded on ICE*_bph-sal_*KF716.
