# Supplementary material for: A New ICEclc Subfamily Integrative and Conjugative Element Responsible for Horizontal Transfer of Biphenyl and Salicylic Acid Catabolic Pathway in the PCB-Degrading Strain Pseudomonas stutzeri KF716
Source: Microorganisms. 2021 Nov 29;9(12):2462. doi: 10.3390/microorganisms9122462 (PMC8704644; doi:10.3390/microorganisms9122462)
Supplement: Supplementary file 1 [file microorganisms-09-02462-s001.zip › microorganisms-1466426-supplementary.pdf]

Table S1 PCR primers used in this study

Primer	Sequence
attL1	5’-GTGTATGCCTTGCCAGTCGCCTTG-3’
attL2	5’-CGCTACCATCAGGTCAAACGCATG-3’
attL3	5’-GGAATTCCACACCATCCGCAATGCC-3’
attR1	5’-CACCGATGCTCTCTACCTACCTGC-3’
attR2	5’-GACATGATCGCCAACACCTCACCG-3’
attR3	5’-CAGTCAGCCAGCAGGCGAGTCTTC-3’
27F	5’-AGAGTTTGATCATGGCTCAG-3’
907R	5’-CCGTCAATTCATTTGAGTTT-3’
bphL1	5’-CGAGAGTCATGTGCCTGAAACC-3’
bphR1	5’-GGTCCAGTACTGGGTGACCTTG-3’
salL1	5’-ATTCTCCGTGGTGCTACGCGAAGC-3’
salR1	5’-GTCCTTAGCCAGCCAGGTCACCG-3’
MDL1	5’-GCCGAAGCGCTTGGTGAGGTTTC-3’
MDR1	5’-GATGGAGAACATGCGCCTGTATG-3’

Table S2 Genes encoded on $ICE_{bph\text{-}sal}$KF716

Locus	Direction	Start	End	Length	Gene	Product
KF716ICE_10	+	1	18	18	*attL*	
KF716ICE_20	+	256	2109	1854	*int*	integrase
KF716ICE_30	+	2477	3214	738	*bphR*	transcriptional regulator, GntR family
KF716ICE_40	+	3306	4682	1377	*bphA1*	biphenyl dioxygenase alpha subunit
KF716ICE_50	+	4725	5366	642	*bphA2*	biphenyl dioxygenase beta subunit
KF716ICE_60	+	5404	5823	420		hypothetical protein
KF716ICE_70	+	5898	6227	330	*bphA3*	biphenyl dioxygenase system ferredoxin component
KF716ICE_80	+	6224	7450	1227	*bphA4*	biphenyl dioxygenase system ferredoxin-NAD^+ reductase component
KF716ICE_90	+	7497	8330	834	*bphB*	*cis*-2,3-dihydrobiphenyl-2,3-diol dehydrogenase
KF716ICE_100	+	8352	9248	897	*bphC*	2,3-dihydroxybiphenyl 1,2-dioxygenase
KF716ICE_110	+	9280	9891	612	*bphX0*	glutathione *S*-transferase
KF716ICE_120	+	9974	10756	783	*bphX1*	2-oxopent-2,4-dienoate hydratase
KF716ICE_130	+	10777	11691	915	*bphX2*	acetaldehyde dehydrogenase, acetylating
KF716ICE_140	+	11711	12751	1041	*bphX3*	4-hydroxy-2-oxovalerate aldolase
KF716ICE_150	+	12822	13682	861	*bphD*	2-hydroxy-6-oxo-6-phenylhexa-2,4-dienoate hydrolase
KF716ICE_160	+	13785	15158	1374		long-chain fatty acid transport protein
KF716ICE_170	-	15472	15930	459		IS5/IS1182 family transposase
KF716ICE_180	-	16003	17508	1506		retron-type RNA-directed DNA polymerase
KF716ICE_190	+	18261	19436	1176		IS3 family transposase
KF716ICE_200	-	19838	20740	903	*salR*	LysR-family transcriptional regulator
KF716ICE_210	-	20774	21927	1154		IS3 family transposase
KF716ICE_220	+	22119	23432	1314	*salA*	*p*-hydroxybenzoate hydroxylase
KF716ICE_230	+	23875	24213	339	*salB*	2Fe-2S iron-sulfur cluster binding domain-containing protein
KF716ICE_240	+	24210	25133	924	*salC*	catechol 2,3-dioxygenase
KF716ICE_250	+	25168	26628	1461	*salD*	2-hydroxymuconate semialdehyde dehydrogenase
KF716ICE_260	+	26639	27487	849	*salE*	2-hydroxymuconate semialdehyde hydrolase
KF716ICE_270	+	27499	28284	786	*salF*	2-oxopent-2,4-dienoate hydratase
KF716ICE_280	+	28299	29222	924	*salG*	acetaldehyde dehydrogenase, acetylating
KF716ICE_290	+	29235	30275	1041	*salH*	4-hydroxy-2-oxovalerate aldolase
KF716ICE_300	+	30272	31066	795	*salI*	4-oxalocrotonate decarboxylase
KF716ICE_310	+	31115	31306	192	*salJ*	4-oxalocrotonate tautomerase

Table S2 *Cont.*

Locus	Direction	Start	End	Length	Gene	Product
KF716ICE_320	+	31337	31777	441		heme-binding protein
KF716ICE_330	-	31962	32303	342		mobile element protein
KF716ICE_340	+	32612	34042	1431		D-2-hydroxyglutarate dehydrogenase
KF716ICE_350	-	34356	35392	1037		IS110 family transposase
KF716ICE_360	-	35905	36351	447		hypothetical protein
KF716ICE_370	-	37207	39033	1827	*traI*	relaxase
KF716ICE_380	-	39236	40156	921		nucleotidyl transferase AbiEii/AbiGii toxin family protein
KF716ICE_390	-	40149	40907	759		hypothetical protein
KF716ICE_400	+	41131	41502	372		DUF3742 family protein
KF716ICE_410	-	41510	43033	1524	*traG*	conjugal transfer protein TraG
KF716ICE_420	-	43049	43402	354		hypothetical protein
KF716ICE_430	-	43399	44805	1407		integrating conjugative element protein
KF716ICE_440	-	44816	45760	945		TIGR03756 family integrating conjugative element protein
KF716ICE_450	-	45757	46206	450		TIGR03757 family integrating conjugative element protein
KF716ICE_460	-	46357	46851	495		DNA repair protein RadC
KF716ICE_470	-	47047	47790	744		DsbA family protein
KF716ICE_480	-	47804	50692	2889	*virB4*	type IV secretory pathway, VirB4 component
KF716ICE_490	-	50692	51126	435		TIGR03751 family conjugal transfer lipoprotein
KF716ICE_500	-	51107	52543	1437		TIGR03752 family integrating conjugative element protein
KF716ICE_510	-	52533	53447	915		TIGR03749 family integrating conjugative element protein
KF716ICE_520	-	53444	54136	693		TIGR03746 family integrating conjugative element protein
KF716ICE_530	-	54133	54543	411		TIGR03750 family conjugal transfer protein
KF716ICE_540	-	54556	54924	369		TIGR03745 family integrating conjugative element membrane protein
KF716ICE_550	-	54947	55186	240		TIGR03758 family integrating conjugative element protein
KF716ICE_560	-	55183	55554	372		candidate type III effector Hop protein
KF716ICE_570	+	55949	56152	204		VrlI protein
KF716ICE_580	+	56159	56773	615		DUF1819 superfamily putative inner membrane protein
KF716ICE_590	+	56791	57381	591		DUF1788 family protein
KF716ICE_600	+	57411	59567	2157		uncharacterized protein sporadically distributed in bacteria and archaea

Table S2 *Cont.*

Locus	Direction	Start	End	Length	Gene	Product
KF716ICE_610	+	59539	63621	4083		uncharacterized protein sporadically distributed in bacteria and archaea, PglZ domain
KF716ICE_620	+	63636	65705	2070		uncharacterized protein sporadically distributed in bacteria and archaea, not a Lon-type protease
KF716ICE_630	+	65990	67375	1386		hypothetical protein
KF716ICE_640	-	67377	68126	750		TIGR03747 family integrating conjugative element membrane protein
KF716ICE_650	-	68123	70279	2157	*virD4*	Type IV secretory pathway, VirD4 component
KF716ICE_660	-	70288	70821	534		integrating conjugative element protein
KF716ICE_670	-	70818	71438	621		soluble lytic murein transglycosylase and related regulatory proteins
KF716ICE_680	-	71423	72142	720		TIGR03759 family integrating conjugative element protein
KF716ICE_690	-	72229	72876	648		hypothetical protein
KF716ICE_700	-	72873	73484	612	*pilL*	PilL protein
KF716ICE_710	+	73842	74153	312		transcriptional regulator
KF716ICE_720	+	74146	75057	912		transposase
KF716ICE_730	+	75296	77059	1764		DUF2326 domain-containing protein
KF716ICE_740	+	77096	79624	2529		tetratricopeptide repeat family protein
KF716ICE_750	-	79791	82073	2283		superfamily II DNA/RNA helicases, SNF2 family
KF716ICE_760	-	82173	82478	306		hypothetical protein
KF716ICE_770	-	82580	83689	1110		hypothetical protein
KF716ICE_780	-	83755	84405	651		hypothetical protein
KF716ICE_790	-	84490	84855	366		hypothetical protein
KF716ICE_800	-	84945	85556	612		DUF3275 family protein
KF716ICE_810	-	85611	86162	552		DUF3577 domain-containing protein
KF716ICE_820	-	86507	86869	363		hypothetical protein
KF716ICE_830	-	86936	87736	801		hypothetical protein
KF716ICE_840	-	88026	88742	717		hypothetical protein
KF716ICE_850	-	88993	89385	393		hypothetical protein
KF716ICE_860	-	89411	89647	237		hypothetical protein
KF716ICE_870	-	90426	92441	2016		DNA topoisomerase III
KF716ICE_880	-	92736	93119	384		single-stranded DNA-binding protein
KF716ICE_890	-	93116	93664	549	*inrR*	integrase regulator R

Table S2 *Cont.*

Locus	Direction	Start	End	Length	Gene	Product
KF716ICE_900	-	93661	94449	789		TIGR03761 family integrating conjugative element protein
KF716ICE_910	-	94638	95390	753		plasmid stabilization system protein
KF716ICE_920	-	95629	96822	1194		hypothetical protein
KF716ICE_930	-	96826	97386	561		DUF2857 domain-containing protein
KF716ICE_940	-	97404	99047	1644	*parB*	chromosome partitioning protein ParB
KF716ICE_950	-	99040	99276	237		hypothetical protein
KF716ICE_960	-	99260	100126	867	*parA*	ParA family protein
KF716ICE_970	-	100167	100385	219		AlpA family phage regulatory protein
KF716ICE_980	-	100510	101265	756		receptor protein-tyrosine kinase
KF716ICE_990	-	101820	102731	912		LysR family transcriptional regulator
KF716ICE_1000	+	103073	103734	662		hypothetical protein
KF716ICE_1010	-	103745	104218	474		putative transcription regulator protein
KF716ICE_1020	-	104267	105466	1200		putative outer membrane protein
KF716ICE_1030	-	105463	106329	867		hypothetical protein
KF716ICE_1040	-	106329	107738	1410		metallo-beta-lactamase family protein, RNA-specific
KF716ICE_1050	-	107746	107949	204		hypothetical protein
KF716ICE_1060	-	107970	109508	1539		outer membrane factor OMF1 associated with YbhGFSR efflux transporter
KF716ICE_1070	-	109505	110623	1119		ABC-type efflux pump permease component YbhR
KF716ICE_1080	-	110628	111797	1170		ABC-type efflux pump permease component YbhS
KF716ICE_1090	-	111794	113566	1773		ABC-type efflux pump, duplicated ATPase component YbhF
KF716ICE_1100	-	113568	114608	1041		ABC-type efflux pump membrane fusion component YbhG
KF716ICE_1110	-	114636	115055	420		hypothetical protein
KF716ICE_1120	-	115246	115854	609		transcriptional regulator, AcrR family
KF716ICE_1130	+	116138	117034	897		putative transcriptional regulatory protein
KF716ICE_1140	+	117283	117300	18	*attR*	
